# A case of suspected paraneoplastic nerve syndrome associated with prostate cancer or opsoclonus‐myoclonus syndrome associated with COVID‐19 infection, but symptoms improved after treatment of both

**DOI:** 10.1002/iju5.12825

**Published:** 2025-01-02

**Authors:** Naoya Tomomasa, Sotaro Kayano, Tatsuya Monzen, Tatsuya Shimomura, Takahiro Kimura

**Affiliations:** ^1^ Department of Urology SUBARU Health Insurance Society Ota Memorial Hospital Ota Gunma Japan; ^2^ Department of Urology Jikei University School of Medicine Minato‐Ku Tokyo Japan; ^3^ Department of Neurology SUBARU Health Insurance Society Ota Memorial Hospital Ota Gunma Japan

**Keywords:** opsomyoclonus, paraneoplastic neurological syndromes, prostate cancer

## Abstract

**Introduction:**

Paraneoplastic neurological syndrome is a type of neurological syndrome that occurs in patients with cancer; however, it is rarely associated with prostate cancer. We herein report a rare case of this condition.

**Case presentation:**

A 76‐year‐old man, treated conservatively for rotatory vertigo due to a subtype of Guillain–Barré syndrome after COVID‐19, was referred to our neurology department. Magnetic resonance imaging showed no obvious findings; however, paraneoplastic neurological syndrome was suspected due to opsomyoclonus, and close examination of the primary tumor revealed a high prostate‐specific antigen level and bone metastases, suggesting prostate cancer. Paraneoplastic nerve syndrome associated with prostate cancer was suspected. Since the possibility of OMS associated with COVID‐19 infection was considered, bilateral orchiectomy and endocrine therapy, as well as pulse steroid therapy, were performed, and the patient's symptoms resolved.

**Conclusion:**

This rare case suggests the need for timely and aggressive treatment for prostate cancer–associated paraneoplastic nerve syndrome.

**Abbreviations & Acronyms**
CSF: cerebrospinal fluid
MRI: magnetic resonance imaging
OMS: opsoclonus‐myoclonus syndrome
PNS: paraneoplastic neurological syndrome

**Keynote message**

PNS associated with prostate cancer is extremely rare. We encountered an exceptionally rare case in which PNS was suspected owing to unexplained rotatory vertigo, and the symptoms improved with therapeutic intervention. When PNS is suspected, timely and aggressive treatment is necessary.

## Introduction

Paraneoplastic neurological syndrome (PNS) is caused by immunological mechanisms and occurs in patients with cancer. PNS is relatively rare, with an incidence of approximately 0.01%–1% among all patients with malignant tumors; PNS associated with prostate cancer is particularly rare.^1^ Herein, we describe a rare case of suspected PNS associated with prostate cancer wherein the patient's symptoms improved after therapeutic intervention.

## Case presentation

**Fig. 1:**
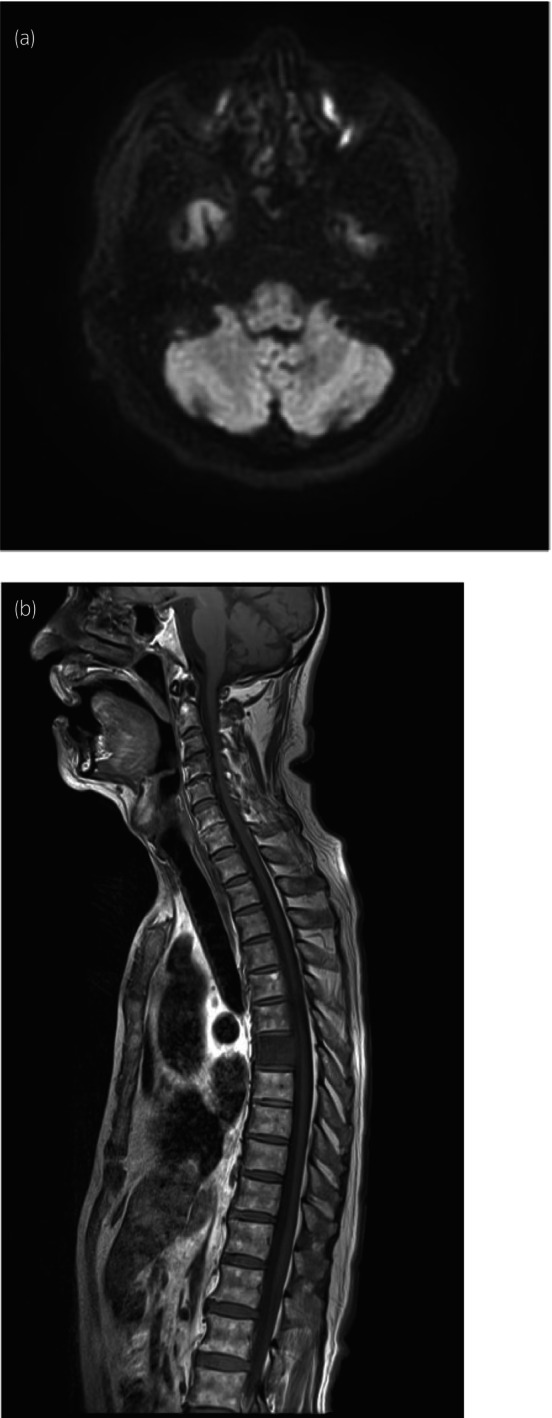
MRI upon close inspection. (a) MRI of the head showing no obvious abnormal findings. (b) MRI of the spinal cord showing no obvious abnormal findings.

The patient was a 76‐year‐old male who initially developed a cough and high‐grade fever, based on which he was diagnosed with COVID‐19. However, 8 days after the diagnosis of COVID‐19, rotatory dizziness and nausea appeared, and he was diagnosed with peripheral vertigo. Ten days later, an eye movement disorder was observed. At that time, the patient was conscious and had rotatory vertigo and right horizontal nystagmus, which worsened with body movement. Magnetic resonance imaging (MRI) of the head revealed no abnormalities (Fig. 1a). However, cerebellar symptoms subsequently developed. Suspecting spinocerebellar disease, MRI of the spinal cord was performed but showed no abnormal findings (Fig. 1b), while cerebrospinal fluid (CSF) examination showed no obvious abnormal findings. The patient was treated conservatively for a subtype of Guillain–Barré syndrome caused by COVID‐19 infection. However, the symptoms showed no improvement, and he was referred to our neurology department for further evaluation.

**Fig. 2:**
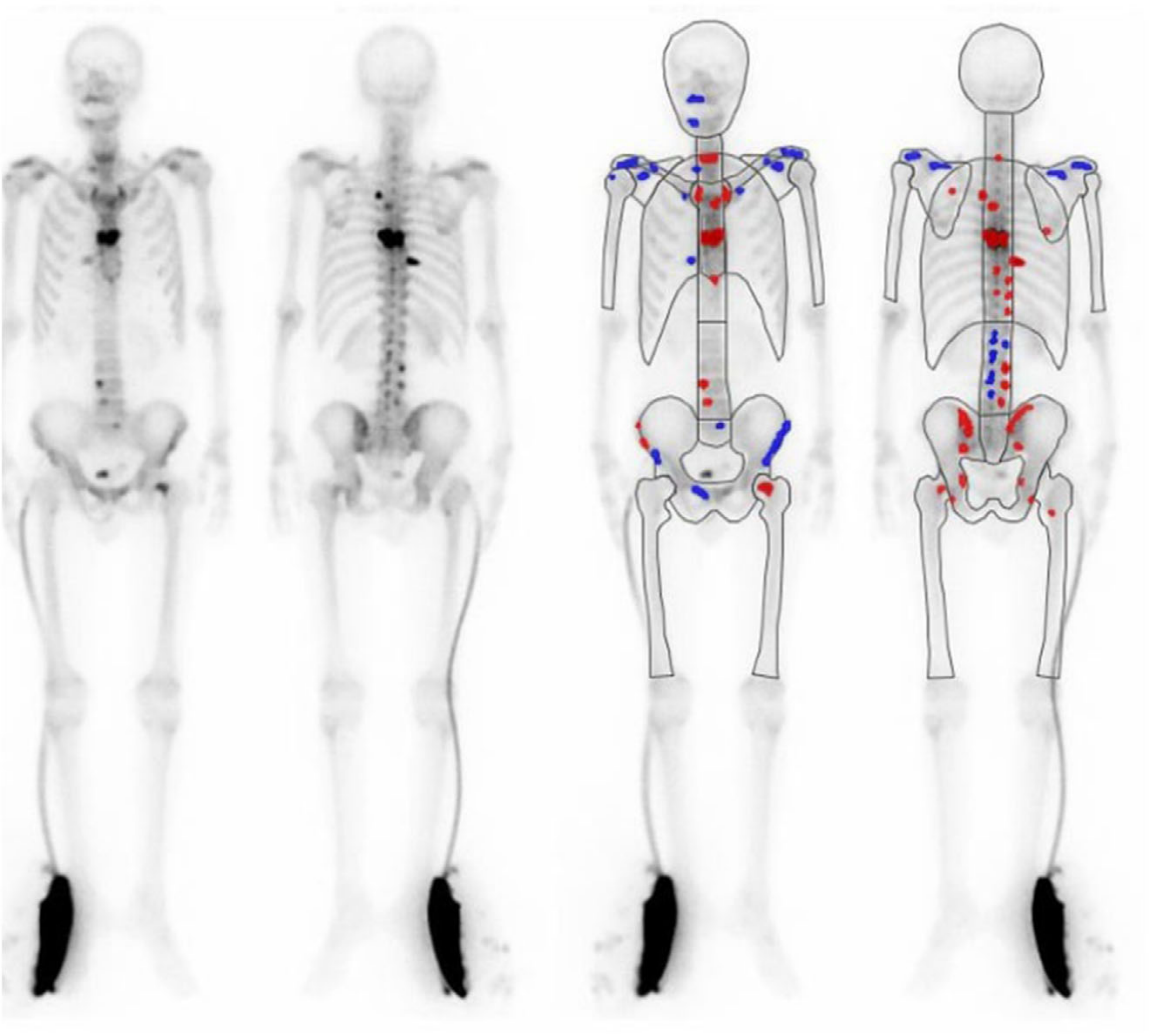
Bone scintigraphy shows findings of bone metastases.

Physical examination by a neurologist suggested that opsomyoclonus findings (random eye movements and trunk myoclonus) were the main cause of the symptoms. Therefore, we suspected a diagnosis of PNS and assessed tumor markers and performed scintigraphy; high PSA levels (249.95 ng/mL), and positive Ga scintigraphy (suspected bone metastasis of Th4 and 7), and bone scintigraphy (32 hot spots with bone scan index 1.68%) were detected (Fig. 2). Another differential diagnosis considered was the possibility of OMS associated with COVID‐19 infection.

The neurologist initiated steroid pulse therapy for PNS and OMS associated with COVID‐19 infection, and we initiated endocrine therapy (degarelix acetate and bicalutamide) for prostate cancer. Two weeks later, a prostate biopsy and bilateral orchiectomy (because we strongly suspected prostate cancer and sustained outpatient visits may be difficult in the future, we opted for an orchiectomy) revealed prostate cancer with a Gleason score of 4 + 5 (all 6 positive cores). Although the vertigo and dysarthria gradually improved because of steroid pulse and endocrine therapy, the neurological symptoms did not completely resolve. PSA gradually decreased from 249.95 to 4.04 ng/mL after 3 months of endocrine therapy. Thus, in this case, PNS associated with prostate cancer or OMS associated with COVID‐19 infection was suspected due to unexplained vertigo and neurological symptoms, which improved after treatment.

## Discussion

PNS is observed in small cell lung cancer, breast cancer, and renal cell carcinoma; however, PNS associated with prostate cancer is extremely rare.^1^, ^2^ It affects the central and peripheral nervous systems and neuromuscular junctions, causing various symptoms such as encephalomyelitis, cerebellar degeneration, and dermatomyositis. To date, 34 cases of PNS associated with prostate cancer have been reported, with cerebellar degeneration and encephalomyelitis being particularly common. However, only a few cases reported the involvement of opsomyoclonus.^1^

PNS is associated with antibodies against neural antigens, and anti‐tumor antibodies. Although 62% of PNS cases with prostate cancer are antibody positive, with anti‐Hu antibodies being the most common,^1^, ^3^ the anti‐tumor antibodies were negative in this case. Instead opsoclonus‐myoclonus syndrome (OMS) findings specific to PNS were observed, and the patient was diagnosed with classic neurological syndrome. OMS is significantly less frequent, with only 1% of PNS cases in a recent, representative population‐based epidemiological study.^4^ The Euronetwork criteria for diagnosing PNS have been used since 2004,^5^ which state that for classic neurological syndrome, a malignancy detected within 5 years is a definite diagnosis of PNS. However, in 2021, the diagnostic criteria were updated, and the certainty of PNS diagnosis was divided into three levels,^6^ the most important one stating that the score was influenced by evidence of anti‐tumor antibodies in addition to the existing high‐risk neurological phenotype (Table 1). Therefore, a definitive diagnosis for cases negative for anti‐tumor antibodies is difficult because the PNS Care Score does not exceed 8. Similarly, in this case, the patient had a PNS Care Score of 7, increasing the certainty of the diagnosis.

**Table 1:** PNS care score. In this case, the diagnosis was “probable” with a score of 7^5^

PNS score	Main evaluation item	Sub‐evaluation item	Score
	Clinical level	High‐risk phenotypes	3
		Intermediate‐risk phenotypes	2
		No phenotypes	0
	Laboratory level	High‐risk antibody	3
		Intermediate‐risk antibody	2
		Lower risk antibody or negative	0
	Cancer	Found, consistent with phenotype and (if present) antibody, or not consistent but antigen expression demonstrated	4
		Not found (or not consistent) but follow‐up <2 years	1
		Not found and follow‐up ≥2 years	0
	Diagnostic level	Definite ≥8	
		Probable 6–7	
		Possible 4–5	
		Non‐PNS ≤3	

To the best of our knowledge, only four cases of OMS associated with prostate cancer have been reported. Additionally, a retrospective study analyzed 97 patients diagnosed with PNS using the 2004 diagnostic criteria, of whom 41 (42.3%) had a definite diagnosis whereas 40 (41.2%) were reported as probable using the 2021 diagnostic criteria.^7^ Thus, the new diagnostic criteria have increased diagnostic specificity and decreased sensitivity. Therefore, we recommend timely and aggressive treatment for patients with a probable diagnosis. In this case, MRI excluded an organic lesion, and the disease progressed; therefore, we initiated therapeutic intervention. Moreover, PNS associated with prostate cancer has several histological types, the most common type being small cell carcinoma. Although the tumor was an adenocarcinoma in this case, 35% of cases of PNS are not adenocarcinomas. Moreover, some cases have reported dedifferentiated adenocarcinoma resulting in a small cell carcinoma; therefore, caution is warranted.

The present case also had a history of COVID‐19 infection, and the possibility of OMS associated with COVID‐19 infection was considered.

In fact, there have been reports of OMS as an accompanying symptom of COVID‐19 infection. Other causes of OMS include autoimmune, infectious, toxic, and metabolic diseases.^8^

In terms of treatment for PNS, first‐line immunotherapy involves the administration of intravenous steroids, immunoglobulins, or plasma exchange. Similar immunotherapy is the first‐line treatment for OMS associated with COVID‐19 infection.^8^ If first‐line therapy is unsuccessful, rituximab, cyclophosphamide, mycophenolate mofetil, or azathioprine is used.^9^ Although treatment of the malignancy alone may provide some relief in a few cases, concomitant immunotherapy may be required, as in this severe case.^10^ Moreover, the prognosis of PNS with OMS is often poor with or without immunotherapy, with most patients achieving only partial recovery and others developing severe encephalopathy or coma and succumbing to the disease within a few weeks.^11^ Of the four reported cases of OMS in prostate cancer, three had poor prognosis^12^, ^13^, ^14^ (Table 2). Moreover, only one case was positive for anti‐Hu and anti‐Yo antibodies. Although the prognosis of neurological symptoms generally depends on the degree of cancer progression, the neurological symptoms may not improve even in cases where the prostate cancer has not progressed.^15^ Similarly, in this case, dysarthria improved after therapeutic intervention; however, complete remission was not achieved. Therefore, steroid pulse therapy was completed in three courses, and endocrine therapy was continued. Thus, our report highlights the need for timely and aggressive treatment in case of suspected PNS.

**Table 2:** Comparison of patients with prostate cancer and opsoclonus‐myoclonus syndrome (OMS)^10^, ^11^, ^12^

Reference	Age (years)	iPSA	Anti‐tumor antibodies	Pathology	Metastasis
1	69	14	Negative	Adenocarcinoma	Pelvic bone
2	69	N/D	anti‐Hu antibody and anti‐Yo antibody positive	Small cell carcinoma	Ilium
3	66	N/D	Negative	Adenocarcinoma	Large pelvic lymph node
4	71	N/D	Negative	Adenocarcinoma	Retroperitoneal lymph node
Our case	76	249.95	Negative	Adenocarcinoma	Multiple bone metastases

## Conclusion

We encountered a case wherein PNS associated with prostate cancer was suspected; however, a definitive diagnosis was not obtained, and both prostate cancer and neurological symptoms improved with timely therapeutic intervention.

## Author contributions

Naoya Tomomasa: Writing – original draft. Sotaro Kayano: Writing – review and editing. Tatsuya Monzen: Writing – review and editing. Tatsuya Shimomura: Supervision. Takahiro Kimura: Supervision.

## Conflict of interest

Takahiro Kimura is a paid consultant or advisor for Astellas, Astra Zeneca, Bayer, Janssen, and Sanofi.

## Approval of the research protocol by an Institutional Review Board

Not applicable.

## Informed consent

We obtained consent from the patients for publication.

## Registry and the Registration No. of the study/trial

Not applicable.

## Data Availability

This is an open access article under the terms of the Creative Commons Attribution License, which permits use, distribution, and reproduction in any medium, provided the original work is properly cited.
